# From Rabl-like Architecture to Chromosome Territories: A Conserved Developmental Transition in Animal Genomes

**DOI:** 10.1093/molbev/msaf235

**Published:** 2025-09-22

**Authors:** Jing Liu

**Affiliations:** Women's Hospital, School of Medicine, Zhejiang University, 1 Xueshi Road, Shangcheng District, Hangzhou 310006, China; Evolutionary & Organismal Biology Research Center, School of Medicine, Zhejiang University, 866 Yuhangtang Road, Hangzhou 310058, China

**Keywords:** chromosome territory, Rabl-like, embryonic development, subcompartment

## Abstract

In eukaryotes, chromosomes can be arranged with centromeres and/or telomeres clustered at opposite poles of the nucleus (Rabl-like, RBL) or as discrete spatial domains (chromosome territory, CT). These patterns were thought to be species specific, possibly linked to the presence or absence of condensin II subunits. Time-series embryonic chromatin maps from three distantly related animals reveal that RBL and CT reflect cell state rather than species identity, and a conserved RBL-to-CT transition has occurred during development. This shift is not significantly correlated with condensin II gene expression, but may be influenced by the genomic distribution of repetitive sequences. In late developmental stages, long-range cis- and trans-interactions between active A1 subcompartments progressively intensify, ultimately establishing CT as the dominant architecture. These results uncover a conserved, developmentally regulated reorganization of 3D genome structure across animals.

## Introduction

The nonrandom folding and position of chromosomes within the nucleus play a critical role in genome replication and function. One such organizational feature is the Rabl chromatin architecture, where centromeres and telomeres are clustered at the opposite sides of the nuclear periphery. First identified in salamander cells by Carl Rabl (Rabl 1885), this configuration has since been observed across a wide range of species, including yeast, some plants, and animals (Cremer et al. 1982; Hochstrasser et al. 1986; Haaf and Ward 1995; Abranches et al. 1998). A recent analysis of eukaryotic species’ high-throughput chromatin conformation capture (Hi-C) data defined a broader though somewhat ambiguous term, Rabl-like (RBL) architecture. This refers to the clustering of centromeres and/or telomeres (Hoencamp et al. 2021) and parallelly arranged chromosomes. By contrast, chromosome territory (CT) lacks obvious clustering of centromeres/telomeres and shows major interactions between loci on the same chromosome but not between chromosomes, indicative of more condensed and spatially segregated chromosomes within the nucleus (Cremer and Cremer 2001). Differences between species in these two types of chromosome architectures have been suggested to associate with species-specific presence or absence of condensin II subunits, whose deficiency in human cells transforms the CT into Cen-Cen architecture (Hoencamp et al. 2021). Other evidence from plants (Sakamoto et al. 2022) and *Drosophila* (Bauer et al. 2012) also supports the notion that condensin II plays an important role in preventing centromere clustering (Cen-Cen) during mitosis, either by promoting lengthwise compaction of individual chromosomes or by mediating interactions between centromeres and the nuclear envelope. However, this idea is challenged by observations in some species, which possess intact condensin II units yet exhibit a dominant Rabl organization (Hoencamp et al. 2021), e.g. Western clawed frogs (Bredeson et al. 2024).

Despite its prevalence among eukaryotes, the biological significance of RBL architecture remains largely unclear. In *Drosophila*, Cen-Cen was shown to be critical for the proper silencing of pericentric heterochromatin (Padeken et al. 2013), and the varying tendencies of Cen-Cen in different human chromosomes could influence their interphase positioning in different cell types (Tjong et al. 2016). However, studies in *Arabidopsis thaliana* and human cells have shown that disruptions of nuclear envelope proteins or condensin II have little impact on genome-wide gene expression or the finer structure of compartments and loops (Hu et al. 2019; Hoencamp et al. 2021; Municio et al. 2021). These findings suggest that chromosome organizations may be more flexible than previously thought. Notably, RBL configuration has been observed in mouse embryos before the establishment of CTs, specifically prior to the 64-cell stage (Kitanishi et al. 2022). This observation aligns with previous research showing that a loose chromatin organization is crucial for maintaining embryonic totipotency (Xu and Xie 2018; Zhu et al. 2021). Thus, embryonic development provides an ideal system for studying chromosomal architecture transitions and their underlying functions.

This study analyzes chromatin sequencing data from several model organisms at different embryonic stages, reveals a transition from RBL-to-CT architecture, and proposes that RBL may represent a permissive state followed by the establishment of A1 subcompartmentalization suggestive of progressing cell differentiation. Overall, this study provides new insights into the dynamics and functional roles of chromosomal architecture.

## Results

### A Dynamic Transition of Rabl-like to CT During Development

**Fig. 1.**
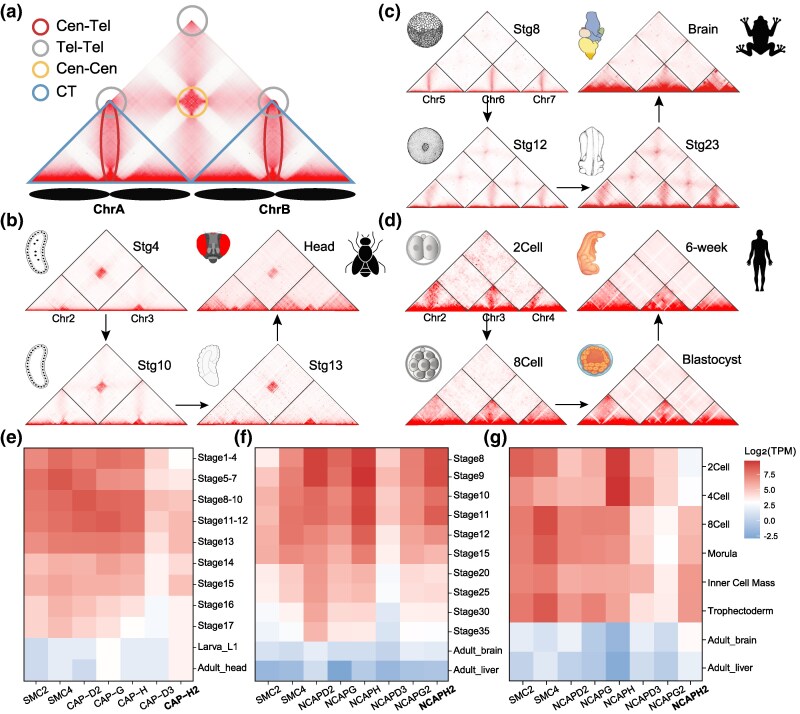
The dynamic of nuclear architecture across embryonic stages of three model species. a) Schematic representations for different architectures, including Cen-Cen, Tel-Tel, and Cen-Tel. The CT is not as clear as others. b to d) Interaction matrices of two or three metacentric chromosomes across the developmental stages of three species. Stronger interactions are indicated by higher signal intensity. e to g) The log2 scaled expression (transcript per million) of condensin II genes in embryonic stages and adult tissues of *D. melanogaster*, *X. tropicalis*, and human.

Previous work (Hoencamp et al. 2021) characterized the chromosomal architectures in postembryonic samples of eukaryotic species. Among them, they observed dominant signals of Cen-Cen for *Drosophila melanogaster* larvae, centromere-to-telomere axis (Cen-Tel) signals in *Xenopus tropicalis* fibroblast cells, and CT in human GM12878 cells. However, nuclear architecture is not constant across cell types or developmental stages, that the configuration observed in a single differentiated cell type may not fully capture the dynamic or polymorphic nature of chromosomal organization in a species. In this study, an integrated Hi-C analysis of chromosomal architecture was performed spanning early to late embryonic stages for these three species (Table S1). Following previous classifications (Hoencamp et al. 2021), this work defines RBL architecture (Fig. 1a) as a configuration exhibiting at least one type of Cen-Cen, telomere clustering (Tel-Tel), and Cen-Tel, which show significant co-occurrence (Hoencamp et al. 2021), distinguishing from CT. In early developmental stages, e.g. stages 4 to 10 of *Drosophila*, all three species displayed a typical Rabl signal, an intrachromosomal (cis*-*) “wings” perpendicular to the main diagonal, along with interchromosomal (trans*-*) interactions between centromeres and/or telomeres (Fig. 1b to d; Figure S1). As development progresses, a general transition from RBL-to-CT configuration can be observed in all three species (Fig. 1b to d; Figure S1). The dynamic was further quantified using the architectural scores (Table S2) by aggregate chromosome analysis (ACA) (Hoencamp et al. 2021). Generally, the scores of three RBL architecture decreased, while the CT score progressively increased during development of three species, albeit with species-specific dynamic (Figure S2). Specifically, the Rabl configuration persisted until the middle-embryonic stage (stage 10; stomodeum invaginates) of *D. melanogaster* and was dominated by CT signal afterward (Fig. 1b; Figure S1). Notably, strong Cen-Cen interactions remained detectable even in adult head tissue when territory-like features are established (Fig. 1b). This observation suggests that Rabl-like and CT architectures are not strictly mutually exclusive but rather undergo a dynamic and gradual reorganization. In *X. tropicalis*, the Cen-Tel and Cen-Cen configuration remained strong until late-stage 23 (early tailbud stage) (Fig. 1c; Figure S1), whereas in human embryos, the Rabl configuration dissolved rapidly, and CT emerged as early as eight-cell stage (Fig. 1d; Figure S1).

### Factors that Determine the Architectural Transition

Previous studies have shown that disruption of the condensin II subunit (*NCAPH2*) in human cells results in a transition from CT to RBL architecture (Hoencamp et al. 2021). However, the clawed frog has a fully functional condensin II yet exhibits persistent RBL architecture. To examine whether condensin II gene expression directly correlates with chromosomal architecture during development, *CAP-H2* expression patterns were analyzed across the three species. Interestingly, *CAP-H2* expression did not correlate with CT signals in any species (Fig. 1e to g; Figure S3). This suggests that condensin II is not the sole determinant of RBL or CT architecture and that other factors could have contributed to the transitions under the context of embryonic development.

Computational simulations predict that chromatin architecture is shaped by lengthwise compaction, heterochromatin phase separation, and nuclear envelope attachment (Brahmachari et al. 2022). These processes are closely linked to repetitive elements (Lu et al. 2021), particularly pericentromeric and telomeric repeats. I therefore hypothesize that genomic distribution of repetitive elements can influence architectural transitions (Figure S4). In *D. melanogaster* and *X. tropicalis*, the extended maintenance of RBL architecture during embryogenesis (relative to humans) may be driven by phase separation between large pericentromeric and telomeric heterochromatin (Larson et al. 2017; Strom et al. 2017), reflected by the higher density of repeats in *Drosophila*'s (peri-)centromeric (∼20.0% of entire chromosomes) (Hoskins et al. 2002) and frog's (peri-)centromeric and telomeric regions (∼47.3%) (Bredeson et al. 2024) compared to flanking arms (Figure S4). Consistently, both species still exhibit visible signals of Cen-Cen in adult tissue (Figure S1). By contrast, in humans, dense repeats along chromosomal arms may interfere with or overwhelm the interactions of centromeric/telomeric repeats, favoring CT formation (Krijger and de Laat 2013). Further investigation across a broader range of species is required to conclude.

### Architectural Transitions Are Implicated in A1 Sub-compartmentalization

**Fig. 2.**
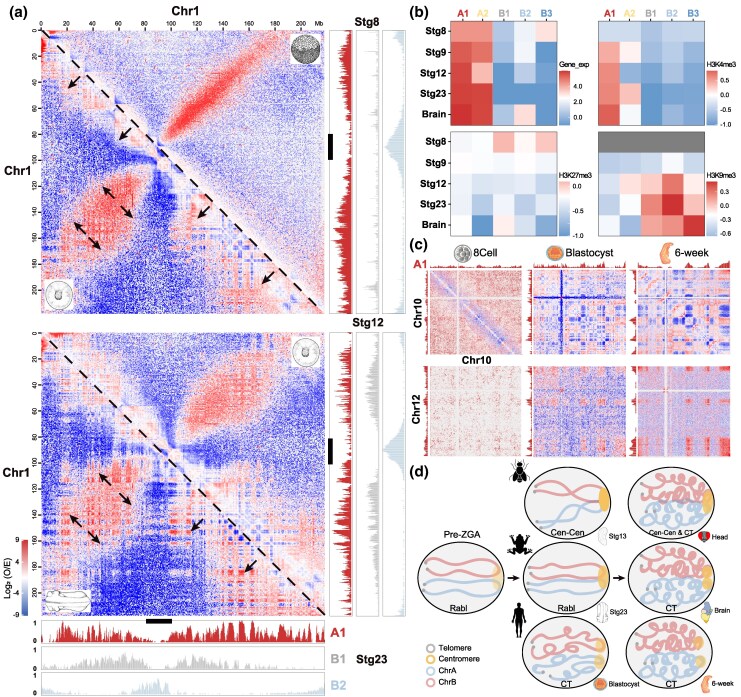
The changes of intra- and interchromosomal interactions across *X. tropicalis* and human development. a) The intrachromosomal O/E interaction matrix of Chr1 from right upper to left below represents the sequential comparisons from Stg8 to Stg12 and Stg12 to Stg23. The red signals mainly reflect enhanced A1–A1 interactions, different in measurement from Cen-Tel-polarized axis. The latter reflects a polarized spatial feature: centromeres and telomeres positioned at opposite nuclear poles and quantified by bin-wise aggregate analysis. The black arrows represent the regions with increased interaction during development, most of which are from A1 subcompartments (red) shown in the attached histograms. The black bars indicate pericentromeric regions. The colored bars represent the score of different subcompartments. b) Heatmaps showing average gene expression and histone modification levels (H3K4me3, H3K27me3, and H3K9me3) across subcompartments (A1, A2, B1, B2, B3; *x* axis) and developmental stages (Stg8, Stg9, Stg12, Stg23, adult brain; *y* axis). Subcompartments A1 and A2 are associated with transcriptional activity and active histone marks (particularly H3K4me3), while B1 is enriched for the polycomb-mediated repression mark H3K27me3. B2 and B3 show low gene expression and are enriched for constitutive heterochromatin features, including H3K9me3 (in earlier stages) and repetitive sequences such as LINEs and satellite DNA. Note that H3K9me3 data in Stg8 and Stg9 were not available (gray boxes). c) The dynamic of subcompartments across early embryonic development of humans on Chr10 and Chr12. The upper panel represents the intrachromosomal interaction of Chr10, and the below panel represents the interchromosomal interaction between Chr10 and Chr12. The A1 subcompartment classification was adjusted according to the GM12878 profiles from Rao et al. (2014). After the blastocyst stage, either the intra- or interchromosomal interaction between A1 subcompartments is progressively increased followed by B1 subcompartments. d) The model of embryonic transition of RBL to CT in three species. At early stages, three species are characterized by RBL structure with Cen-Cen/Tel-Tel and intermingled chromosome arms. The fly and frog persist this structure until late stages with established CT, though Cen-Cen is still observed. In humans, the RBL rapidly disappeared after ZGA. This model suggests a dynamic but conserved pattern of chromosomal organization in distant animal species.

The shift from RBL-to-CT architecture during embryonic development likely reflects a broader transition from a relaxed chromatin state, conducive to totipotency, to a more complex chromatin organization required for cell differentiation (Zhu et al. 2021). In line with this, a gradual increase in long-range (>2 Mb) cis- and trans-interactions during development can be observed (Figures S5 and 6). To examine the functional implications of this transition, Hi-C interaction profiles were integrated with epigenetic histone modification and repeat element profiles in *X. tropicalis* to predict subcompartments (Table S3). They are known to associate with functional subnuclear structures such as nuclear speckle, periphery, and nucleolus (Quinodoz et al. 2018; Spracklin et al. 2023). Consistent with previous studies (Rao et al. 2014; Wang et al. 2021), the highest levels of active histone modification and gene expression were found within A1 subcompartments, followed by A2 (Fig. 2b; Figure S7a). B1 subcompartments, which are established in later stages, were associated with polycomb-repressive complex-related H3K27me3, while B2 and B3 subcompartments correlated with pericentromeric repeats (e.g. LINE CR1 and DNA PiggyBac) and telomeric repeats (e.g. Satellite and DNA hat (Bredeson et al. 2024)), respectively (Figure S7b). These results indicate that the subcompartment classification in this study is robust and corresponds to epigenetic and regulatory alterations.

Visual inspection revealed a significant increase in long-range cis-interactions, particularly within A1 subcompartments, during development (Fig. 2a), in line with previous reports of genome compartmentalization following zygotic genome activation (ZGA) (Niu et al. 2021). The chromatin interactions of A1 subcompartments are then progressively segregated by B1 subcompartments during later developmental stages. Similar, though weaker, A1–A1 trans*-*interactions were also observed, except for dominant centromeric interactions within B2 subcompartments (Figure S8). Most developmentally invariant subcompartments belong to A1, while B1 and B3 were more developmentally specific (Figure S9). Therefore, it is likely that the genome-wide strengthening of A1–A1 interactions (Figure S10), in both long-range cis- and trans-scales, has reinforced the chromatin entanglements, contributing to the overwhelming of CT signals over RBL organization. Supporting this, a strong correlation (Pearson correlation test, *P* = 0.0162) between A1 compartmentalization strength and architectural score of CT can be found, while B2/3 compartmentalization strength is positively correlated with Rabl score in a weak significance (Figure S11). A similar pattern of A1 subcompartmentalization was also observed during human embryonic development (Fig. 2c), yet the B3 subcompartment may have also contributed to the CT architecture, which differs from *X. tropicalis* (Figure S12). Although subcompartment profiles are unavailable in *D. melanogaster*, a notable A-to-B compartment switch was initially observed at stage 13, coinciding with the RBL-to-CT transition (Figure S13). After masking centromeric regions to reduce polarization bias in compartment calling, earlier shifts became apparent, for example on chrX at stage 8 and chr2R at stage 10, suggesting a more gradual compartment formation. To visualize the architectural transitions, 3D chromosome organization in *X. tropicalis* was reconstructed using miniMDS (Rieber and Mahony 2017). In early stages, chromosome trajectories are clear with loose entanglements for both cis- and trans-interactions, and RBL architecture remains persistently strong until the adult brain, where centromere and Tel-Tel dissipated, giving way to distinct chromosome territories (Figure S14).

## Discussion

This work reveals a general transition from RBL-to-CT architecture during embryonic development across distantly related species (Fig. 2d). In pre-ZGA stages, all three species prominently display RBL architecture, which gradually transforms toward CT as embryogenesis proceeds. Ultimately, CT architecture is firmly established in adult tissues or the late embryonic stage (as observed in the 6-week human embryo), although Cen-Cen persists to varying degrees in *D. melanogaster* and *X. tropicalis*. This work suggests that differences in repeat density and distribution along chromosomes may contribute to species-specific transitions. Notably, the role of condensin II in regulating genome architecture (Hoencamp et al. 2021) during embryonic development warrants further investigation.

Beyond repeat distribution and condensin II activity, cell cycle kinetics may also influence architectural transitions. Early embryonic cell cycles are typically rapid, leaving limited time for postmitotic chromatin reorganization (Dernburg et al. 1996). This can result in residual “mitotic memory” in chromatin topology (Hoencamp et al. 2021), favoring RBL features in early stages. Although condensin II may compact chromosomes during mitosis, the abbreviated interphase may be insufficient for fully establishing CT organization. In this kinetic context, RBL architecture might represent a default organizational state during interphase. This raises a reciprocal question: what mechanisms enable some species to form constant CTs? Certain species-specific nuclear features may provide insight. For example, extensive somatic homolog pairing in *Drosophila* (and other dipteran insects) (Hughes et al. 2018; AlHaj Abed et al. 2019; Erceg et al. 2019; Child et al. 2021; Contessoto et al. 2023) could sustain trans-homolog interactions and hinder CT segregation. Besides, chromatin tethering to nuclear landmarks such as the lamina (LADs) or nucleolus (NADs, often forming chromocenters) may restrict chromosomal mobility and promote compartmentalization, thereby reinforcing CT architecture in most mammalian cells. These spatial anchors may also obscure simple Cen-Cen signals in Hi-C, particularly in species with relatively small pericentromeric heterochromatin (PCH) regions, such as humans (8.1% in CHM13.v2 assembly). By contrast, chromosomes of *D. melanogaster* and *X. tropicalis* contain larger PCH (and telomeric heterochromatin) regions (∼20.0% of *Drosophila* and ∼47.2% of frog) and lower repeat density along flanking arms (Figure S4), which may facilitate persistent Cen-Cen and even affect mitotic exit (Santos and Shaw 2004). Collectively, these observations suggest that RBL and CT architectures are not strictly opposing states but rather represent a dynamic continuum, probably shaped by cell type/cycle, architectural proteins, nuclear tethering, heterochromatin phase separation, and genomic repeat landscapes.

What is the biological signiﬁcance of the RBL conﬁguration? Although the RBL configuration may not significantly influence genome-wide gene expression (Wilkie et al. 1999; Hoencamp et al. 2021), chromatin condensation levels do impact chromatin topology and gene transcription (van Steensel and Belmont 2017). The loose chromatin organization in early embryos is essential for totipotency (Xu and Xie 2018; Zhu et al. 2021), and RBL architectures may facilitate effective chromosome mixing (Kitanishi et al. 2022), promoting certain regulatory contacts. The Hi-C analysis in this work demonstrates the gradual establishment of A1 subcompartmentalization as a key feature of the RBL-to-CT architectural transition. This process likely corresponds to the developmental progression of cells from totipotency to functional differentiation. Future studies spanning larger phylogenetic scales and developmental stages, using cutting-edge technologies, e.g. Pore-C (Deshpande et al. 2022; Zhong et al. 2023) and scNanoHi-C (Li et al. 2023), will provide greater power to elucidate the mechanism and function of chromosome architecture shifting.

## Materials and Methods

### Data Collection

A total of 28 Hi-C data across stages or tissues of *D. melanogaster* (Ogiyama et al. 2018; Chathoth and Zabet 2019; Ing-Simmons et al. 2021; Zenk et al. 2021), Western clawed frog (Niu et al. 2021), and human (Schmitt et al. 2016; Chen et al. 2019) (Table S1) were downloaded for analysis. The reference genomes are Flybase r6.39 for *D. melanogaster*, UCB_Xtro_10.0 for *X. tropicalis*, and GRCh38.p14 for human data. The epigenetic data for *X. tropicalis* embryos were downloaded from NCBI GEO dataset: GSE67974 (Hontelez et al. 2015), and gene expression atlas was obtained from Bgee suite (Bastian et al. 2021).

### Chromosome Architecture Analysis

The Cutadaper (v4.4) (Martin 2011) and Trimmomatic (v0.32) (Bolger et al. 2014) were used to remove the adapter and trim raw reads. The clean Hi-C read for each species was mapped to genome assemblies using Bowtie2 (Langmead and Salzberg 2012), with only uniquely mapped reads were kept (mapping quality (MAPQ) ≥ 20), then the binning and normalization were performed by HiC-Pro (v2.10.0) (Servant et al. 2015) with the default parameters at 25, 50, 100, and 500 Kb and 1 Mb resolutions under iterative correction and eigenvector decomposition normalization (Imakaev et al. 2012). The read counts of short-range (<2 Mb) and long-range (≥2 Mb) cis- and trans-interactions were generated with the module “hicTransform” of HiCExplorer (Ramírez et al. 2018). For the visualizations of Hi-C interaction matrix, the software Juicerbox (v2.17) was used with MAPQ ≥ 0. The ACA (Hoencamp et al. 2021) was used to characterize and measure the architectural score of RBL and CT. Specifically, each chromosome was rescaled to a uniform length according to the centromere positions, and then the interaction contacts were aggregated for centromeres, telomeres, and flanking regions for all metacentric or submetacentric chromosomes. The subcompartments (50 Kb) were predicted using software CscoreTool-M (Zheng et al. 2023) and assigned to A1, A2, B1, B2, and B3 types through their correlations with epigenetic modifications and genomic characteristics, e.g. repeat and gene density. To define the subcompartment type for each bin, the A/B compartment eigenvector value was integrated into the subcompartment scores. For human stages, the subcompartment assignment was manually adjusted based on the calling profiles of the GM12878 cell line (Rao et al. 2014).

## Supplementary Material

msaf235_Supplementary_Data

## Data Availability

All datasets analyzed in this study are publicly available. Hi-C datasets for *D. melanogaster*, *X. tropicalis*, and human, as well as epigenetic and gene expression data, are described in the Data Collection section with accession numbers and references provided.
