# Supplementary material for: From Rabl-like Architecture to Chromosome Territories: A Conserved Developmental Transition in Animal Genomes
Source: Mol Biol Evol. 2025 Sep 22;42(10):msaf235. doi: 10.1093/molbev/msaf235 (PMC12532108; doi:10.1093/molbev/msaf235)
Supplement: msaf235_Supplementary_Data [file msaf235_supplementary_data.zip › Supplementary figures.Final_version.pdf]

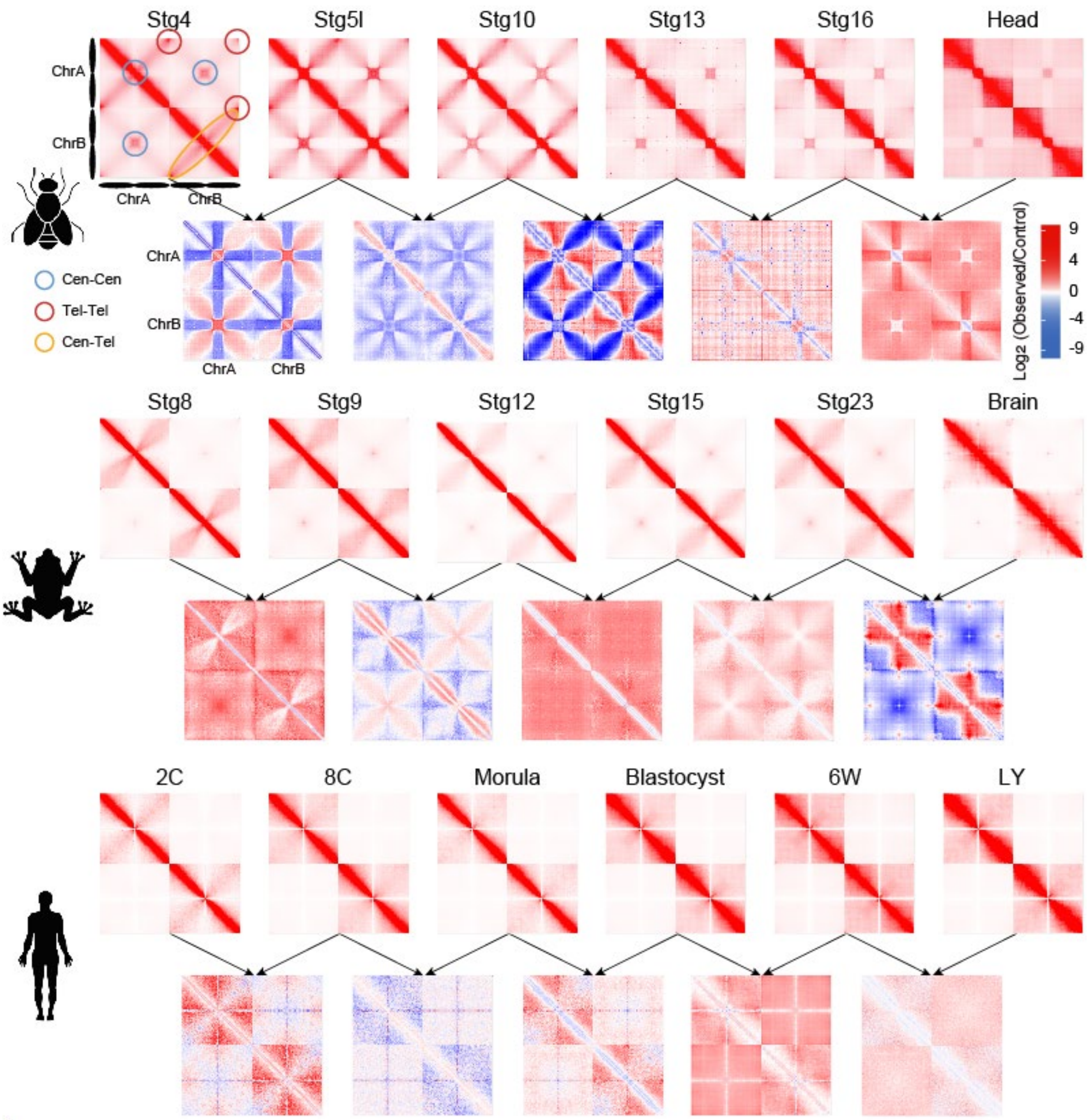

Fig. S1. The dynamic of nuclear architecture across embryonic stages of three model species. Each matrix includes two aggregated chromosomes with centromeres in the middle and telomeres at both ends. The more red color represents stronger interaction (O/E, observed vs expected; Knight-Ruiz normalization). For each species, the subjacent matrix is the comparison of interaction matrices between later stages vs earlier stages on the upper panels. The red color represents increased O/E interaction in later stages, and blue color suggests decreased interaction in later-stages. 2C: 2-cell stages; LY: lymphoblastoid.

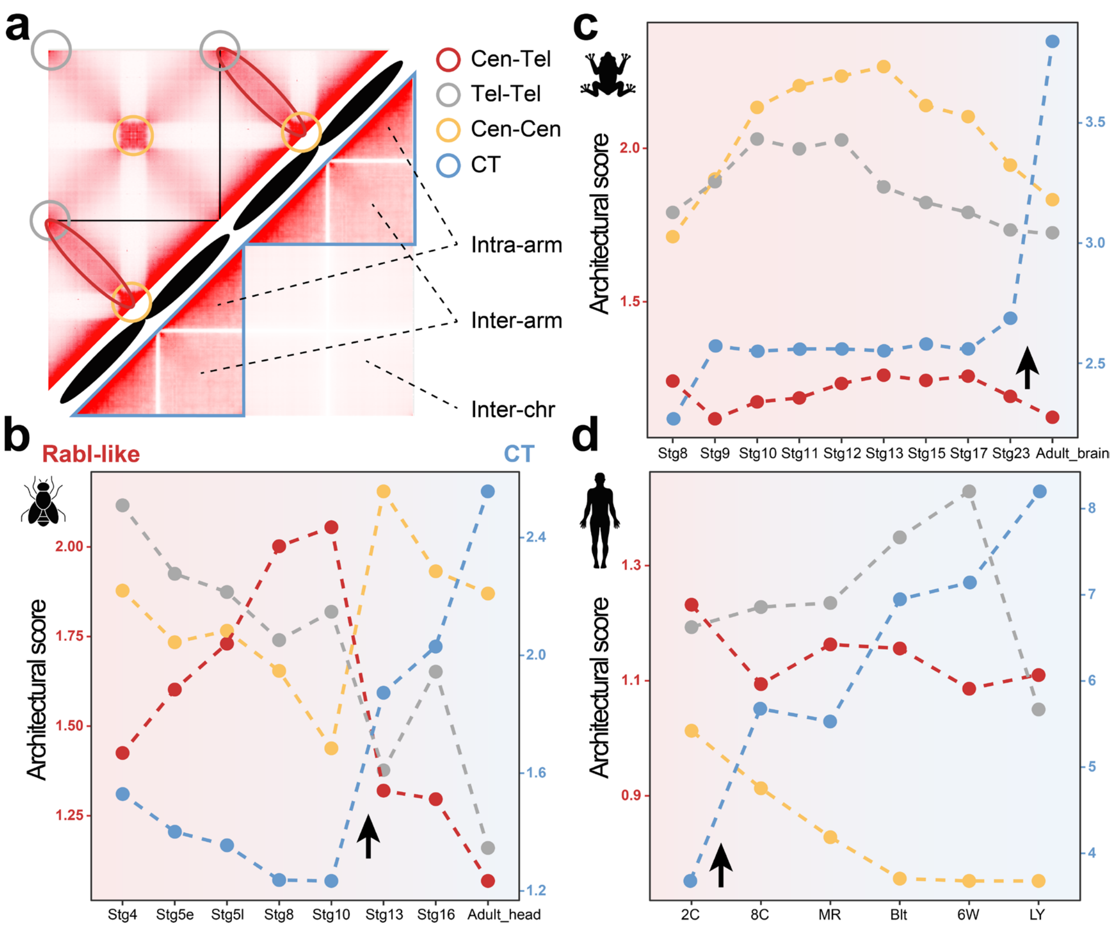

Fig. S2. The dynamic of architectural scores across embryonic stages of three model species. a) The schematic plots for different architectures. **b-d**) The left and right Y-axis in the line-point plots represents the RBL (including Cen-Tel in red, Tel-Tel in gray and Cen-Cen in yellow) and CT (blue) scores, respectively. The black arrows are the stages when the architecture undergoes the largest shifting.

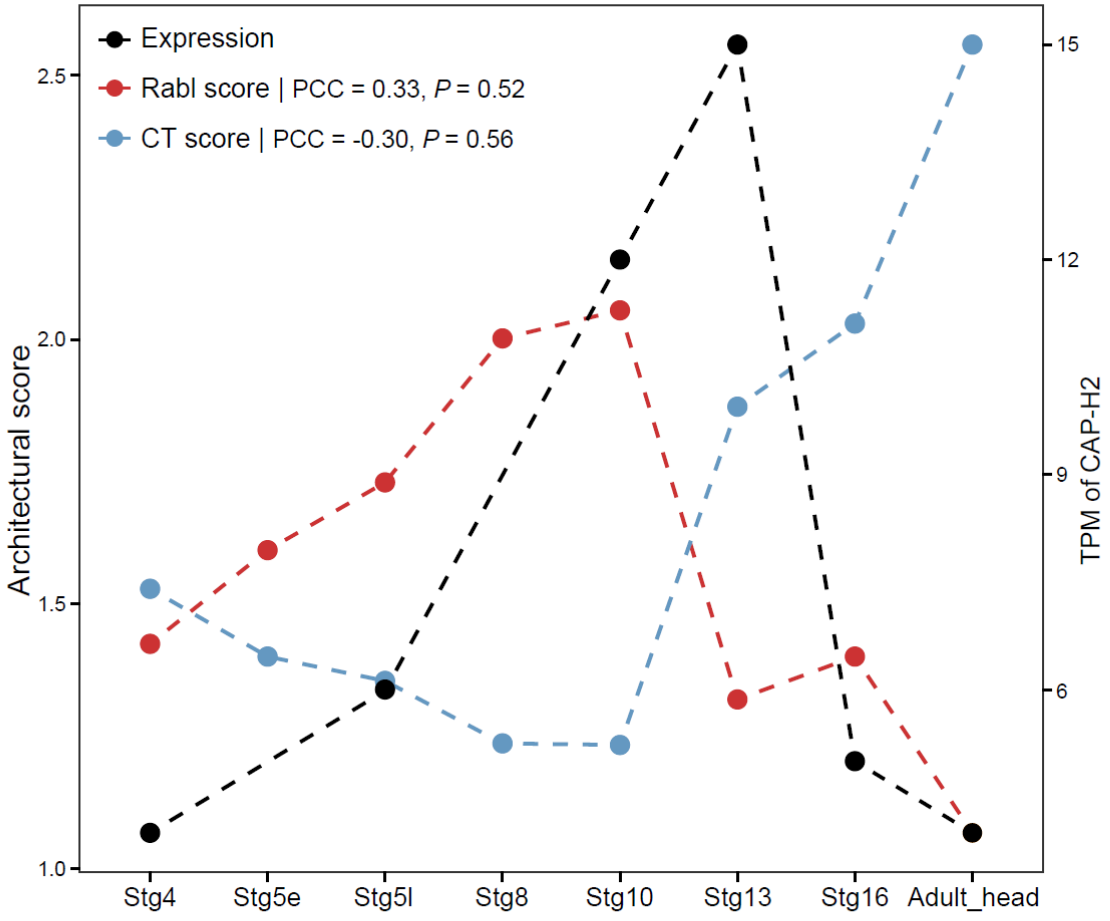

Fig. S3. The expression of *NCAPH2* genes is not coincidental with the CT architectural score across the *Drosophila* development, even exhibiting a contrary pattern although without significance.

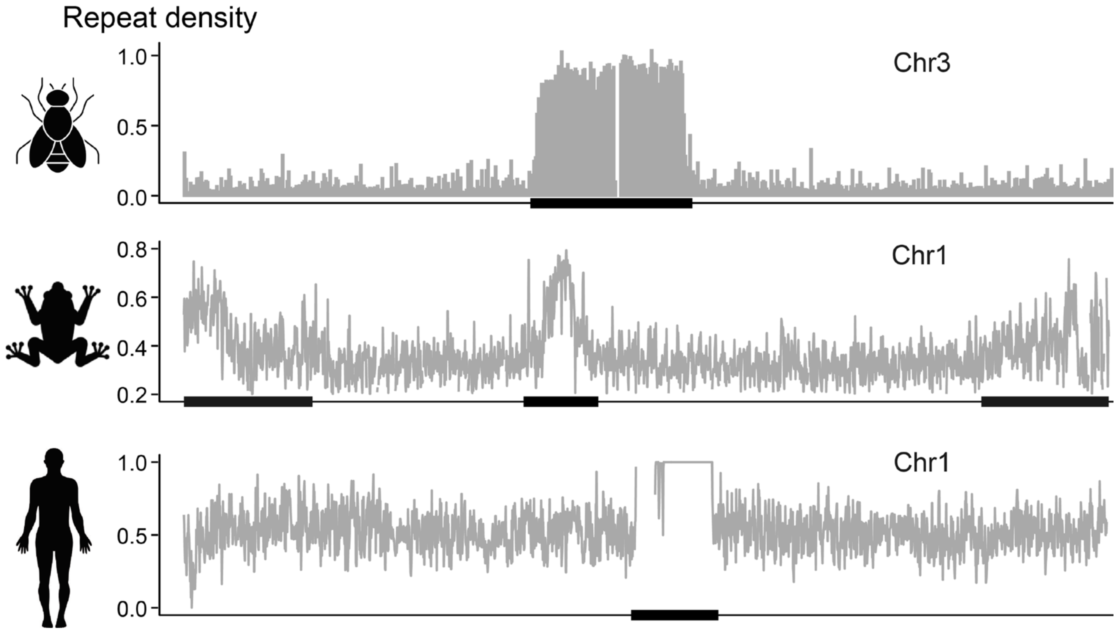

Fig. S4. The repeat landscape might influence the RBL or CT architecture. The plots represent repeat density across the chromosomes of fruit fly, frog and human, The black marks represent pericentromeric or telomeric heterochromatin regions.

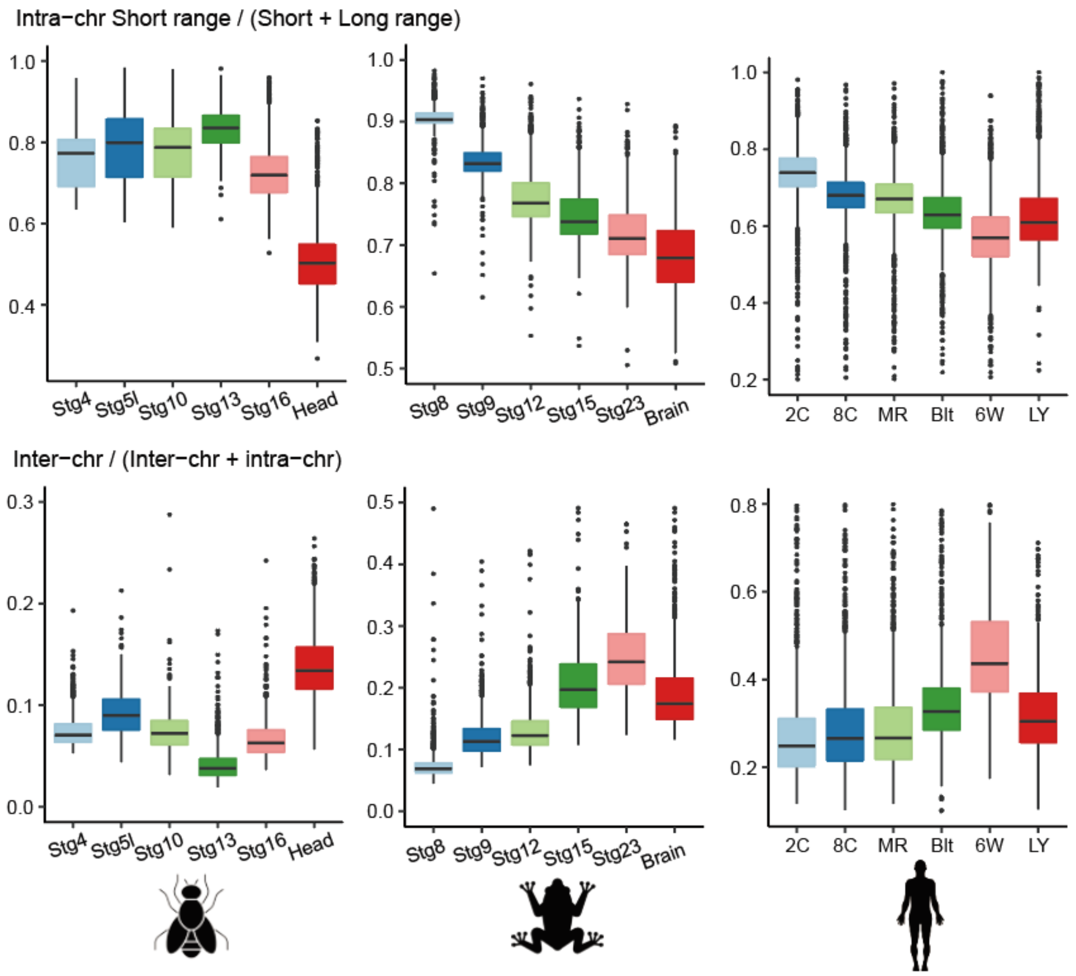

Fig. S5. The summary of interaction contacts in three model species. a) The ratio of intra-chromosomal short vs long range distance interactions across developmental stages and species. The interaction is defined as short-range if the distance between two 100Kb-bins is smaller than 2Mb; **b)** The ratio of inter- vs intra-chromosomal interactions across developmental stages and species.

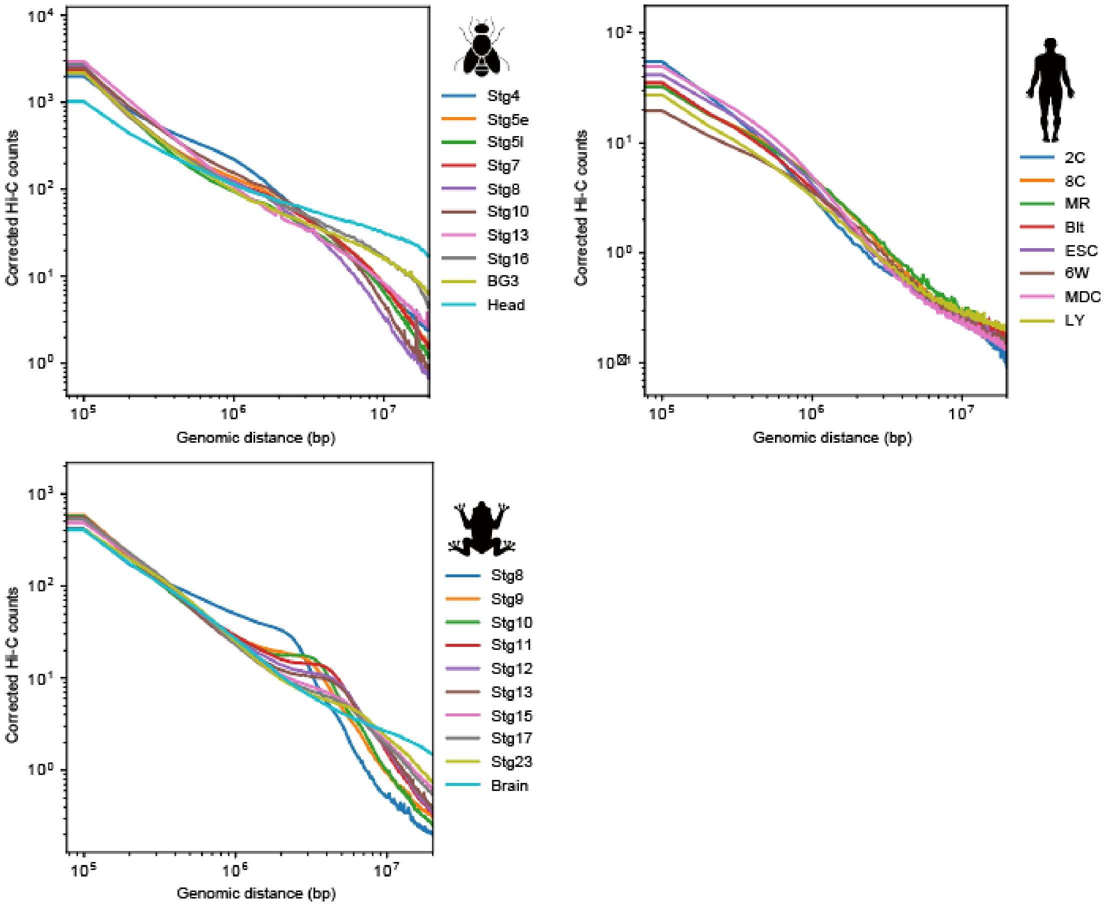

Fig S6. The decay of corrected interaction counts along with the genomic distance across developmental stages and species. The later embryonic stages exhibit more long-range (distance >2Mb) interactions. BG3: cell lines derived from *drosophila* larval central nervous system; MR: morula; Blt: blastocyst; ESC: embryonic stem cell; MDC: myeloid dendritic cell.

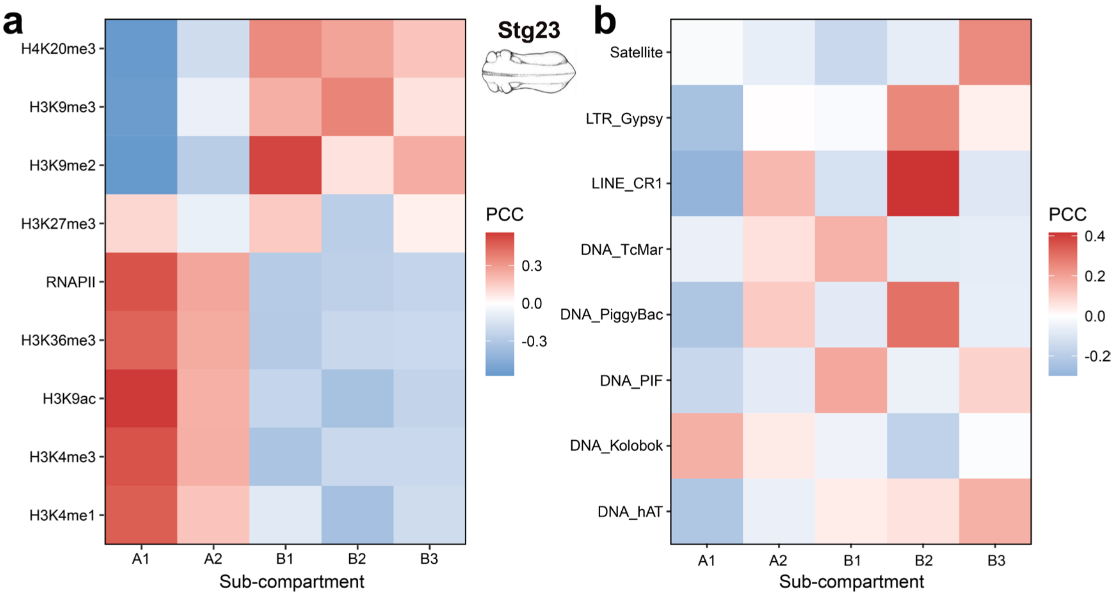

Fig. S7. The correlation of sub-compartments with epigenetic modifications (left) and genomic repeats (right) for frog stage23. a) Pearson correlation coefficients (PCC) between sub-compartments (A1-B3) and various histone modifications or chromatin features. A1 shows the strongest positive correlations with active transcription marks (e.g., H3K4me3, H3K36me3, H3K9ac, RNAPII), followed by A2. In contrast, B sub-compartments are increasingly associated with repressive marks such as H3K27me3, H3K9me2/3, and H4K20me3. **b)** PCC between sub-compartments and major repeat element classes. B2 sub-compartments are enriched for pericentromeric repeats such as LINE_CR1 and DNA_PiggyBac, while B3 shows stronger associations with telomeric repeats including satellite DNA and DNA_hAT.

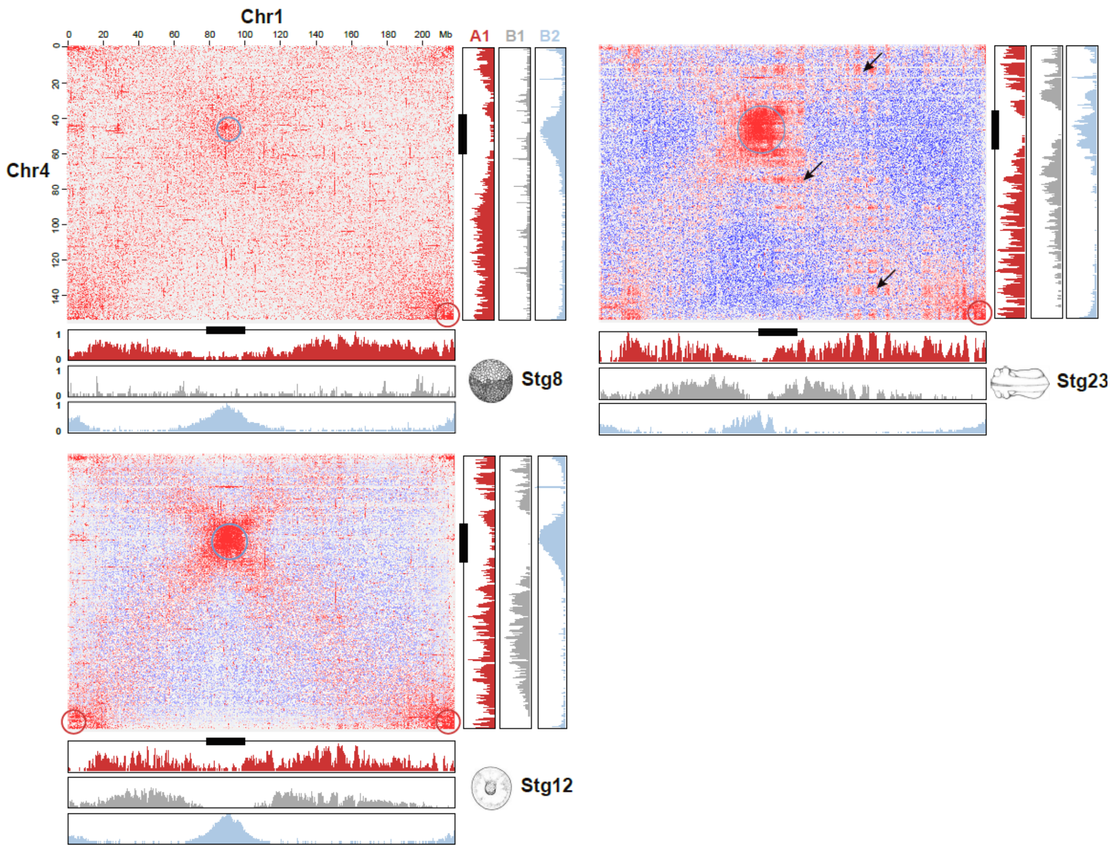

Fig. S8. The inter-chromosomal O/E interaction matrix between Chr1 and Chr4 across the three stages. There are clear inter-chromosomal centromeric interactions (B2 sub-compartments). Except for that, the *trans*-interaction of A1 sub-compartments is weakly established.

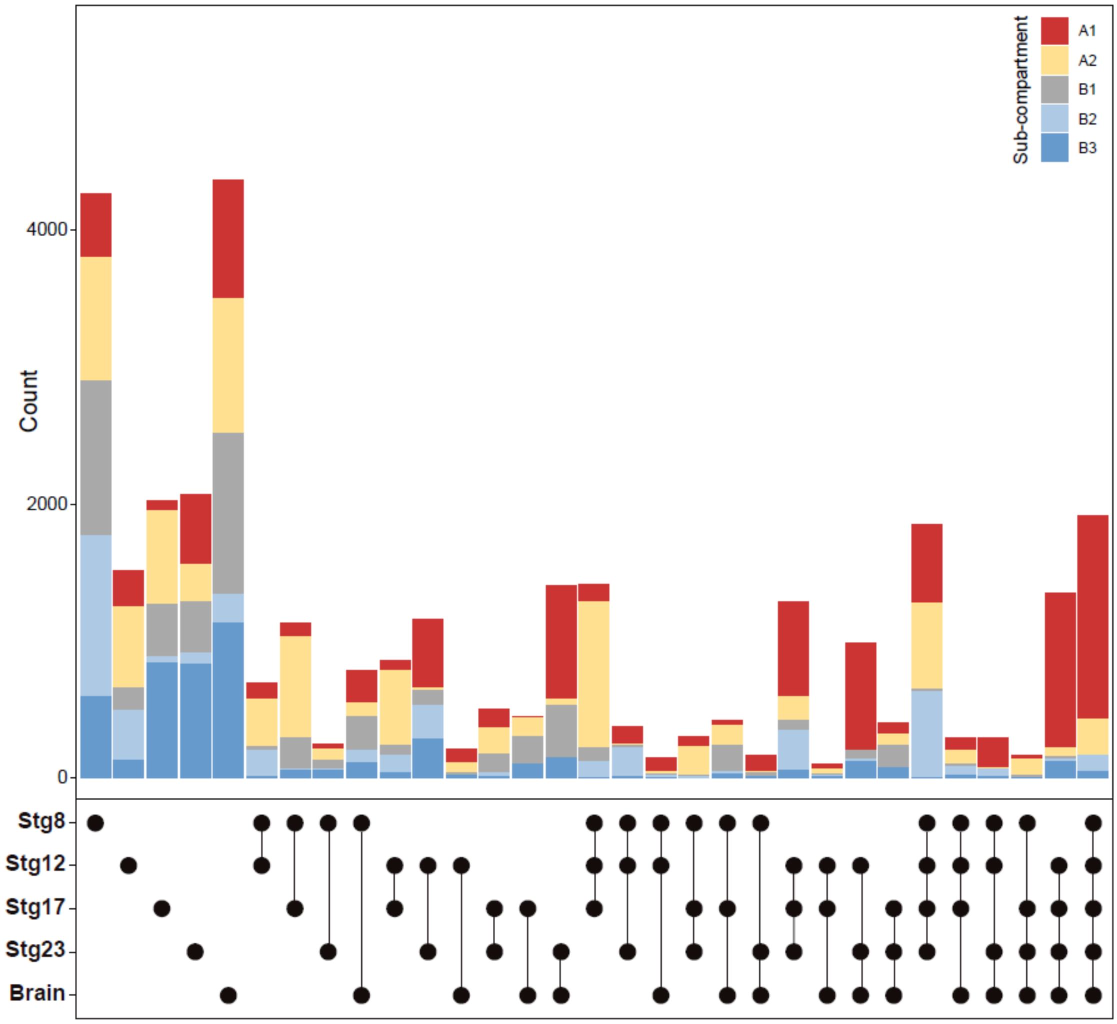

Fig. S9. The overlap numbers of sub-compartments across developmental stages of *X. tropicalis*. For each 50Kb window, we define whether it has an invariant sub-compartment type across stages or stage specific type. It turns out that A1 is the most stable compared to others across stages, while B1/3 is more stage specific.

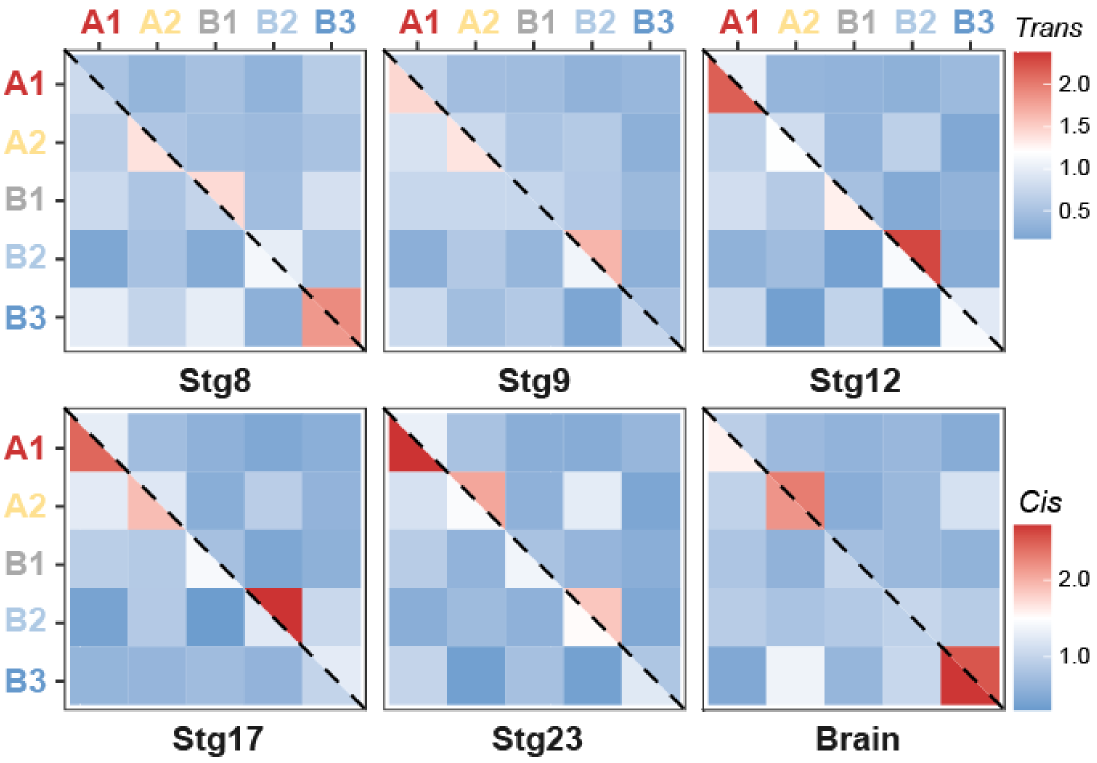

Fig. S10. The *cis*- (left below) and *trans*- (right upper) sub-compartmentalization strength for different sub-compartments across stages in *X. tropicalis*.

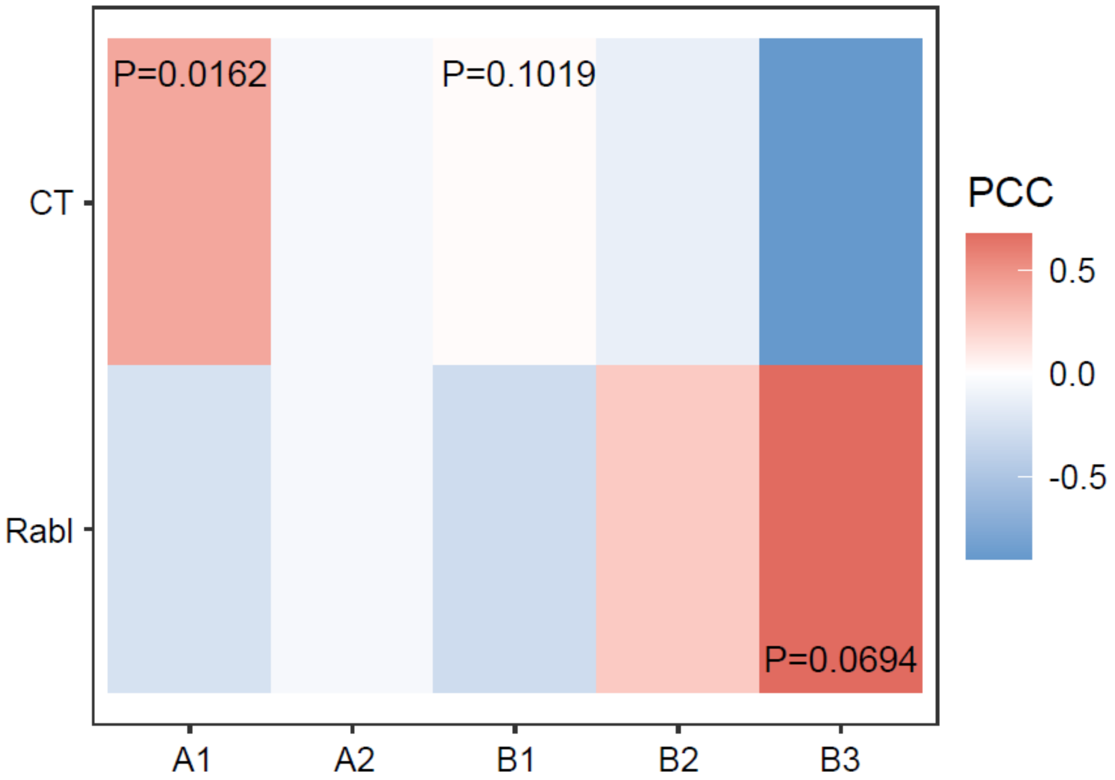

Fig. S11. The correlation of sub-compartmentalization strength with CT and Rabl architectural scores of *X. tropicalis.* The correlation is calculated based on the value of compartmentalization and architectural scores across embryonic stages of stg8, 9, 12, 17, 23, brain. The correlations are not significant (*P*>0.05) except for the A1 compartmentalization strength and CT scores.

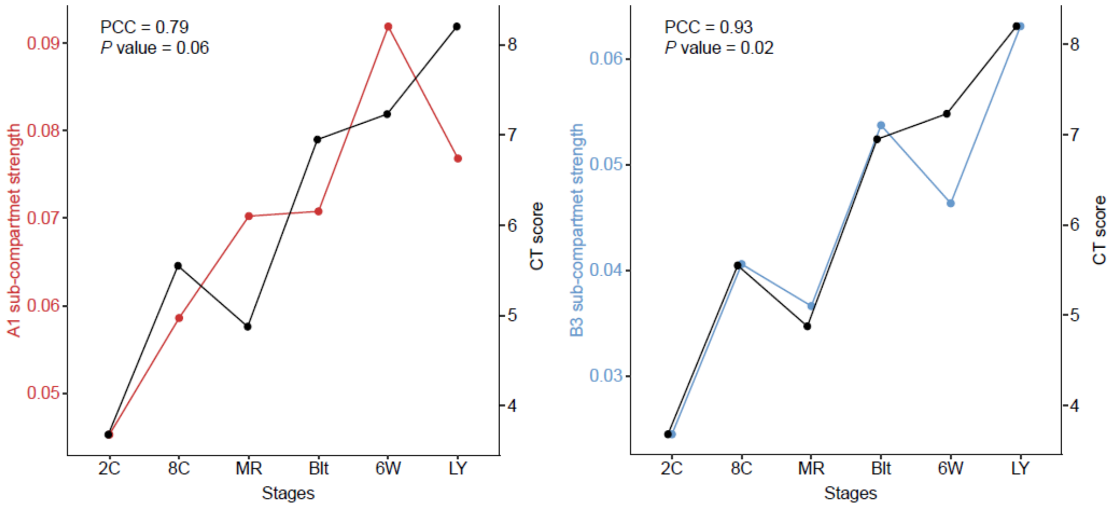

Fig. S12. The correlation of sub-compartmentalization strength with CT architectural scores in human. Only A1 and B3 (in *cis*) show correlation with CT score during development.

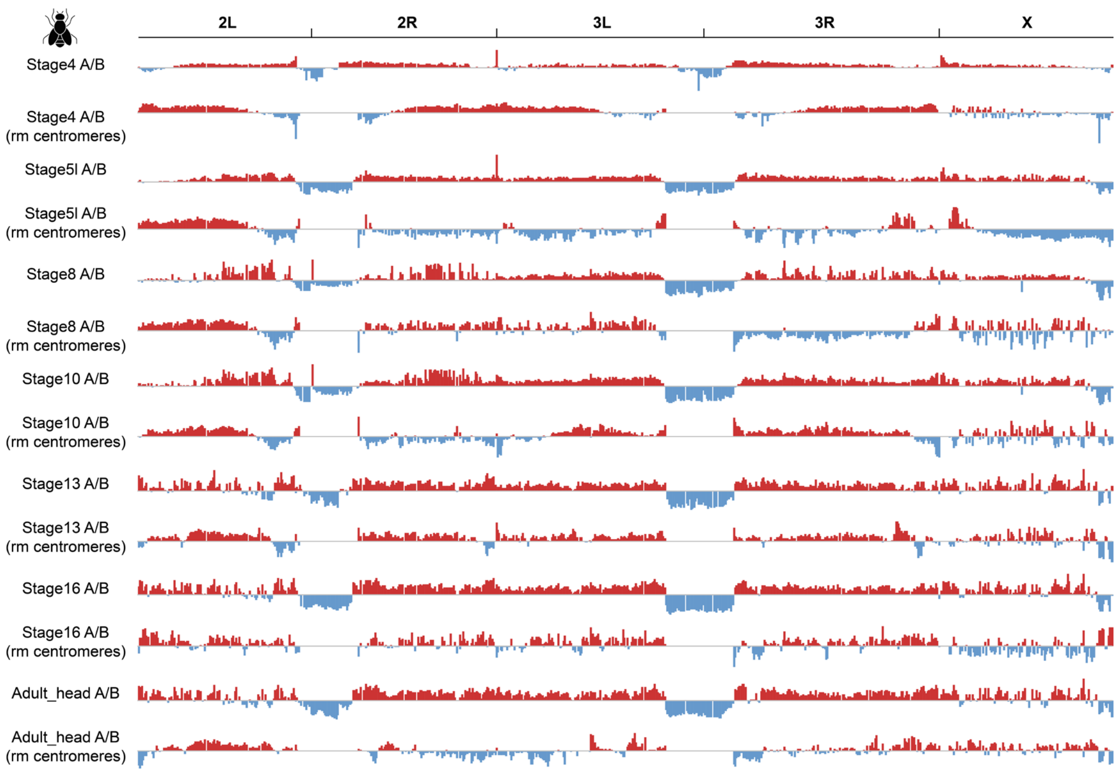

Fig. S13. The compartment landscape of *D. melanogaster* stages. The red color represents the active (A) compartment and blue color represents the inactive (B) compartment. For each stage, the alternative calling of compartments were performed after masking pericentromeric regions.

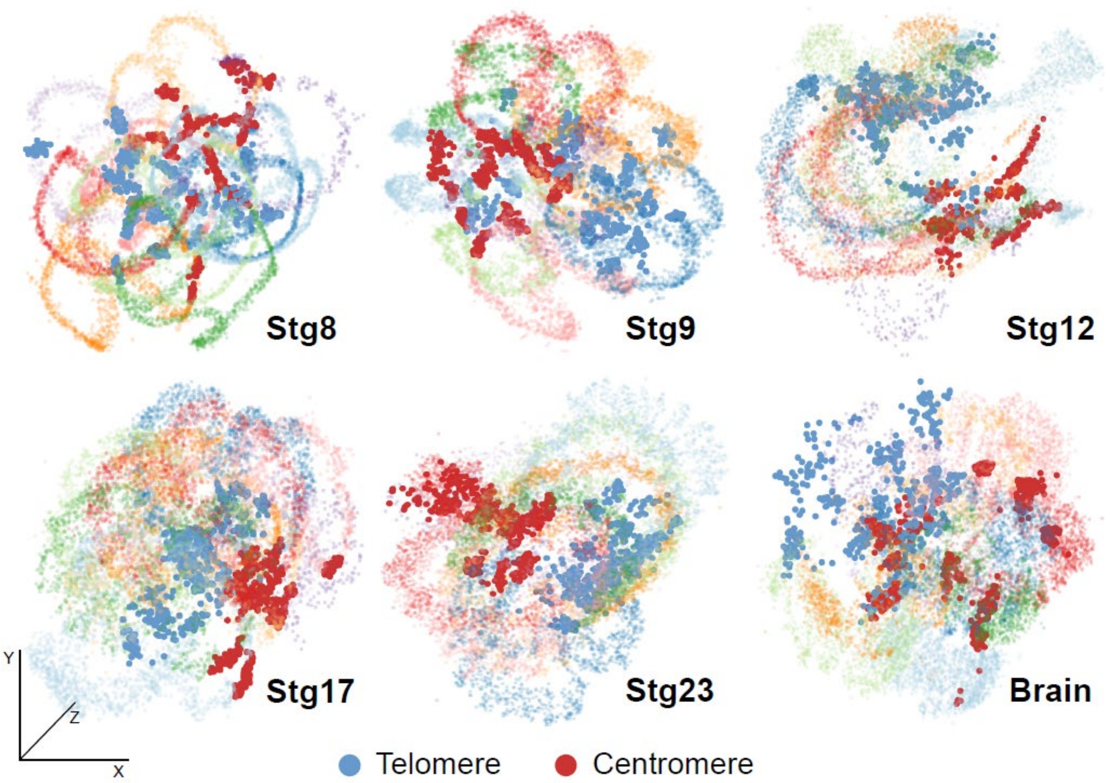

Fig. S14. The reconstructed spatial organizations of frog chromosomes for three stages. Each dot represents one 100Kb bin which belongs to different chromosomes marked with different colors, and the centromeres and telomeres are highlighted by red and blue colors, respectively.
